# A nomogram for predicting survival in patients with skin non-keratinizing large cell squamous cell carcinoma: A study based on the Surveillance, Epidemiology, and End Results database

**DOI:** 10.3389/fmed.2023.1082402

**Published:** 2023-02-17

**Authors:** Jinrong Zhang, Wei Yang, Chengxiang Lian, Qiqi Zhao, Wai-kit Ming, Cheong Cheong Ip, Hsin-Hua Mu, Kong Ching Tom, Jun Lyu, Liehua Deng

**Affiliations:** ^1^Department of Dermatology, The First Affiliated Hospital of Jinan University and Jinan University Institute of Dermatology, Guangzhou, China; ^2^Office of Drug Clinical Trial Institution, The First Affiliated Hospital of Jinan University, Guangzhou, China; ^3^Department of Dermatology, The Fifth Affiliated Hospital of Jinan University, Heyuan, China; ^4^Department of Infectious Diseases and Public Health, Jockey Club College of Veterinary Medicine and Life Sciences, City University of Hong Kong, Hong Kong, Hong Kong SAR, China; ^5^Department of Dermatology, University Hospital Macau, Macau, Macao SAR, China; ^6^General Surgery Breast Medical Center, Taipei Medical University Hospital, Taipei City, China; ^7^Primax Biotech Company, Hong Kong, Hong Kong SAR, China; ^8^Department of Clinical Research, The First Affiliated Hospital of Jinan University, Guangzhou, China

**Keywords:** Surveillance, Epidemiology, and End Results, cancer-specific survival, nomogram, non-keratinizing large cell squamous cell carcinoma, SEER

## Abstract

**Introduction:**

This study aimed to develop and validate a nomogram for predicting cancer-specific survival (CSS) in patients with non-keratinized large cell squamous cell carcinoma (NKLCSCC) at 3, 5, and 8 years after diagnosis.

**Methods:**

Data on SCC patients were collected from the Surveillance, Epidemiology, and End Results database. Training (70%) and validation (30%) cohorts were generated using random selection of patients. Independent prognostic factors were selected using the backward stepwise Cox regression model. To predict the CSS rates in patients with NKLCSCC at 3, 5, and 8 years after diagnosis, all of the factors were incorporated into the nomogram. Indicators such as the concordance index (C-index), area under the time-dependent receiver operating characteristic curve (AUC), net reclassification index (NRI), integrated discrimination improvement (IDI), calibration curve, and decision-curve analysis (DCA) were then used to validate the performance of the nomogram.

**Results:**

This study included 9,811 patients with NKLCSCC. Twelve prognostic factors were identified by Cox regression analysis in the training cohort, which were age, number of regional nodes examined, number of positive regional nodes, sex, race, marital status, American Joint Committee on Cancer (AJCC) stage, surgery status, chemotherapy status, radiotherapy status, summary stage, and income. The constructed nomogram was validated both internally and externally. The nomogram had good discrimination ability, as indicated by the comparatively high C-indices and AUC values. The nomogram was properly calibrated, as indicated by the calibration curves. Our nomogram was superior to the AJCC model, as illustrated by its superior NRI and IDI values. DCA curves indicated the clinical usability of the nomogram.

**Conclusion:**

The first nomogram for prognosis predictions of patients with NKLCSCC has been developed and verified. Its performance and usability demonstrated that the nomogram could be utilized in clinical settings. However, additional external verification is still required.

## Introduction

Cutaneous squamous cell carcinoma (cSCC) is the second most common type of non-melanoma skin cancer. It accounts for 20% of skin cancers, with 1 million cases and an estimated 9,000 deaths each year in the United States (1). The reported incidence of cSCC ranges from 5 to 499 per 100,000 patients (2–5). Among the non-Hispanic white population in the United States, the lifetime risk of developing SCC is 14–20% (6, 7). This risk has continued to increase each year, with an estimated increase of between 50 and 200%, and is likely to continue to increase due to the aging population (8). However, the current understanding of non-keratinized large cell squamous cell carcinoma (NKLCSCC) of the skin is inadequate, and its increasing incidence and distinct characteristics from other types of squamous cell carcinoma require it to be independently analyzed.

Some researchers have proposed that the most significant risk factors for cSCC include age, sex, race, and surrounding environment (9), but there is currently no definite prognostic implication for the subtypes of cSCC. The present study addressed the postoperative recurrence of NKLCSCC, which is one of the most common subtypes of squamous cell carcinoma.

A fundamental standard of care for cancer treatment is the standard American Joint Committee on Cancer (AJCC) staging system. But when used to predict the prognosis of NKLCSCC, the AJCC staging system is restricted by the lack of precise demographic and clinical characteristics. Providing clinicians with convenient and thorough guidance requires more detailed and extensive prediction models.

Nomograms are highly accurate and easy-to-use tools that are based on many types of tumor prediction models (10) and make it possible to estimate the survival probability of a specific patient. Many researchers have established nomograms for various cancers, including of the tonsils, parotid gland, and breast (11–13), but no nomogram specifically designed for NKLCSCC has been developed. Therefore we developed and evaluated a nomogram for NKLCSCC using pertinent data from the Surveillance, Epidemiology, and End Results (SEER) database to further investigate the prognosis factors for NKLCSCC and its specific treatment.

We aimed to develop a comprehensive nomogram for patients with NKLCSCC in the SEER database that accounted for key demographics, clinicopathological features, and therapeutic approaches in addition to some fundamental traits. We analyzed the treatments applicable to these patients. Our novel nomogram can provide clinicians with more-thorough and personalized patient survival predictions, which makes it clinically superior to conventional methods.

## Patients and methods

### Data sources and research factors

Data were filtered and extracted from the SEER database using SEER*Stat software. Part of the SEER database is available to the public, and we requested additional access to the SEER Plus database (14). We collected NKLCSCC cases from the SEER database by adopting the ICD-O-3 (third revision of the International Classification of Diseases for Oncology) histology/behavior code for NKLCSCC (“8072/3: Squamous cell carcinoma, large cell, non-keratinizing, NOS”) and the cases where the site was cutaneous were selected.

We selected several factors that may be relevant to disease prognosis, including age, race, sex, marital status, tumor grade, AJCC stage, income, number of regional nodes examined (RNE), number of positive regional nodes (RNP), summary stage, surgery status, radiotherapy status, and chemotherapy status. Tumor extension, lymph node metastasis, and distant metastasis as assessed using the TNM (tumor, node, and metastasis) staging system are all included in the AJCC staging system. Due to substantial multicollinearity caused by including all of these factors in the analysis, this study only used the AJCC staging system. Cancer-specific survival (CSS) was the outcome variable. Since the SEER database used in this study did not contain any personally identifying information, it was not necessary to obtain patient-informed permission.

**FIGURE 1:**
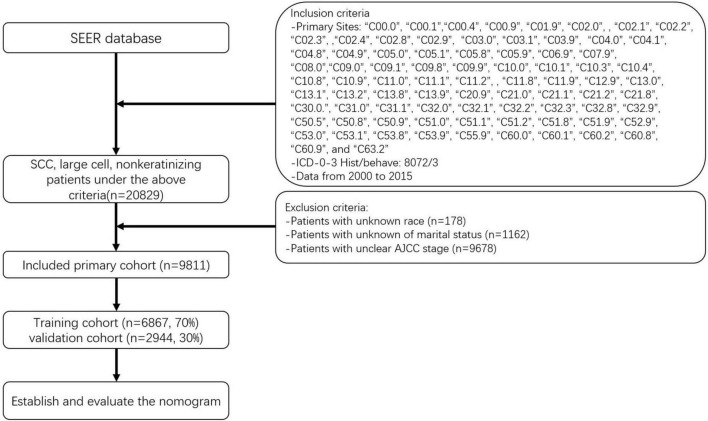
Flowchart of sample selection.

The data of patients whose baseline and survival data were fully available were selected. The seventh edition of the AJCC staging system was adopted. Using the methods described above, we initially identified 20,839 patients with NKLCSCC between 2000 and 2015. After excluding 178 patients with unknown race, 1,162 with unknown marital status, and 9,678 with unknown AJCC stage, 9,811 NKLCSCC patients were finally included (15). These patients were randomly divided into training (70%) and validation (30%) cohorts to test the model using R software (version 4.2.0^1^). Figure 1 illustrates the data screening process.

### Nomogram and statistical analysis

A log-rank test performed after assigning all subjects to the training and validation cohorts indicated no statistically significant differences between them. The initial baseline characteristics of each variable in the study cohort were then summarized using SPSS Statistics software (version 27.0, IBM SPSS, Chicago, IL, United States). Other variable data were presented as frequencies and percentages, while age at diagnosis was expressed as median and interquartile range (IQR) values. Nomograms were utilized to estimate the 3-, 5-, and 8-year CSS probabilities for NKLCSCC, and Cox regression was conducted to identify CSS factors related to NKLCSCC, with a significance cut-off of *p* = 0.05. After constructing the nomogram, we evaluated the model using a set of metrics. Two metrics were used to evaluate the discrimination capability of the nomogram: the concordance index (C-index) and the area under the time-dependent receiver operating characteristic (ROC) curve (AUC). Although AUC and C-index are often utilized, their improvements were not significant relative to the existing model. To ascertain if the new model was superior, we additionally used two relatively new metrics: net reclassification index (NRI) and integrated discriminant improvement (IDI). IDI accounts for numerous tangents that can be utilized to assess the overall performance of the model, and NRI is primarily used to evaluate the prediction capacity of old and new models at a certain tangent level (16, 17). These two markers are better understood in actual clinical applications.

A calibration plot was also constructed to graphically display the variation between the two values. The level of model calibration indicates the consistency of predicted and actual values. When the calibration curve is aligned with the 45° standard line, model consistency has advanced. Decision-curve analysis (DCA) curves were employed to evaluate the clinical validity of the model. The abscissa and ordinate of the DCA curve represent the threshold probability and net profit of the model, respectively. The net profit of a model will be higher if the DCA curve is higher (18).

The R software and SPSS Statistics were used to conduct all statistical analyses. SPSS Statistics were used to characterize the fundamental features of the cohort. R software was then used to randomly divide the data into training and validation cohorts, and a log-rank test was conducted. The following R software packages were used: survival, rms, foreign, survival, survivalROC, nricens, and DCA. These analyses included Cox regression analysis, proportional-hazards regression, nomogram establishment, and assessment. Significance was defined as two-sided probability values of *p* < 0.05.

## Results

### General characteristics

After randomizing 9,811 patients into two cohorts, the log-rank test obtained a probability value (*p* = 1) that indicated no significant differences between them. The fundamental demographic and clinical characteristics of the two cohorts were then described using SPSS (Table 1). The median age at diagnosis was 57 years (IQR = 48–66 years) in the training cohort and 57 years (IQR = 48–65 years) in the validation cohort. The distributions of sex and surgery status were fairly even. Most patients in both the training and validation cohorts were white (78.8 and 79.8%, respectively) and married (56.8 and 55.1%). AJCC stage IV was observed in most cases. Most patients were in the regional cancer summary stage. Most patients were treated with radiotherapy, chemotherapy, and earned US$ 60,000–74,999 per year.

**TABLE 1:** Demographic and clinical characteristics of the two cohorts of patients.

Variable	Training cohort (%)	Validation cohort (%)
*N*	6,867	2,944
Age of diagnosis	57 (48–66)	57 (48–65)
**Sex**		
Male	3,389 (49.4)	1,483 (50.4)
Female	3,478 (50.6)	1,461 (49.6)
**Race**		
White	5,413 (78.8)	2,349 (79.8)
Black	647 (9.4)	247 (8.4)
Other	807 (11.8)	348 (11.8)
**Marital status**		
Single	1,502 (21.9)	655 (22.2)
Married	3,901 (56.8)	1,623 (55.1)
DSW	1,464 (21.3)	666 (22.6)
**AJCC stage**		
I	1,484 (21.6)	610 (20.7)
II	904 (13.2)	396 (13.5)
III	1,583 (23.1)	697 (23.7)
IV	2,896 (42.2)	1,241 (42.2)
**Summary of stage**		
Localized	1,727 (25.1)	723 (24.6)
Regional	3,763 (54.8)	1,632 (55.4)
Distant	1,377 (20.1)	589 (20)
**Radiation**		
Yes	5,318 (77.4)	2,257 (76.7)
No/unknown	1,549 (22.6)	687 (23.3)
**Chemotherapy**		
Yes	4,381 (63.8)	1,868 (63.5)
No/unknown	2,486 (36.2)	1,076 (26.5)
**Income**		
<$35,000, $35,000–$44,999	542 (7.9)	237 (8.1)
$45,000–$59,999	1,456 (21.2)	615 (20.9)
$60,000–$74,999	2,888 (42.1)	1,283 (43.6)
$75,000+	1,981 (28.8)	809 (27.5)
**Surgery**		
Yes	3,166 (46.1)	1,367 (46.4)
No/unknown	3,701 (53.1)	1,577 (53.6)

### Constructing a nomogram using the training cohort

Age at diagnosis, RNE, RNP, sex, race, marital status, AJCC stage, surgery status, chemotherapy status, radiotherapy status, summary stage, and income were the 12 variables that were included after performing multivariate Cox stepwise regression (*p* < 0.05). Table 2 lists the factors that were found to be significant following the multivariate Cox regression analysis, which were age at diagnosis (hazard ratio [HR] = 1.031, *p* < 0.0001), RNE (HR = 0.997, *p* < 0.0001), RNP (HR = 1.003, *p* < 0.0001), sex (HR = 1.237, *p* < 0.0001), black race (versus white: HR = 1.243, *p* < 0.0001), married (versus single: HR = 0.697, *p* < 0.0001), AJCC stage II (versus stage I: HR = 1.429, *p* < 0.0001), AJCC stage III (versus stage I: HR = 1.937, *p* < 0.0001), AJCC stage IV (versus stage I: HR = 2.095, *p* < 0.0001), distant summary stage (versus localized: HR = 2.343, *p* < 0.0001), no/unknown radiotherapy status (versus radiotherapy: HR = 1.702, *p* < 0.0001), no/unknown chemotherapy status (versus chemotherapy: HR = 1.268, *p* < 0.0001), no/unknown surgery status (versus surgery: HR = 1.428, *p* < 0.0001), and income of $75,000+ per year (versus <US$ 35,000 and US$ 35,000–44,999: HR = 0.738, *p* < 0.0001).

**TABLE 2:** Selected variables by multivariate Cox stepwise regression analysis.

	Multivariate analysis		
**Variable**	**HR**	**95% CI**	***p*-Value**
Age of diagnosis	1.031	1.028–1.033	<0.0001
RNE	0.997	0.995–0.998	<0.0001
RNP	1.003	1.002–1.004	<0.0001
**Sex**			
Male	Reference		
Female	1.237	1.154–1.326	<0.0001
**Race**			
White	Reference		
Black	1.243	1.127–1.371	<0.0001
Other	0.977	0.884–1.08	0.6478
**Marital status**			
Single	Reference		
Married	0.697	0.644–0.755	<0.0001
DSW	0.998	0.912–1.092	0.9641
**AJCC stage**			
I	Reference		
II	1.429	1.22–1.674	<0.0001
III	1.937	1.616–2.321	<0.0001
IV	2.095	1.734–2.532	<0.0001
**Summary of stage**			
Localized	Reference		
Regional	1.163	0.995–1.359	0.0572
Distant	2.343	1.968–2.79	<0.0001
**Radiation**			
Yes	Reference		
No/unknown	1.702	1.554–1.865	<0.0001
**Chemotherapy**			
Yes	Reference		
No/unknown	1.268	1.167–1.379	<0.0001
**Income**			
<$35,000, $35,000–$44,999	Reference		
$45,000–$59,999	0.891	0.791–1.004	0.0573
$60,000–$74,999	0.823	0.736–0.919	0.0006
$75,000+	0.738	0.655–0.831	<0.0001
**Surgery**			
Yes	Reference		
No/unknown	1.428	1.317–1.547	<0.0001

**FIGURE 2:**
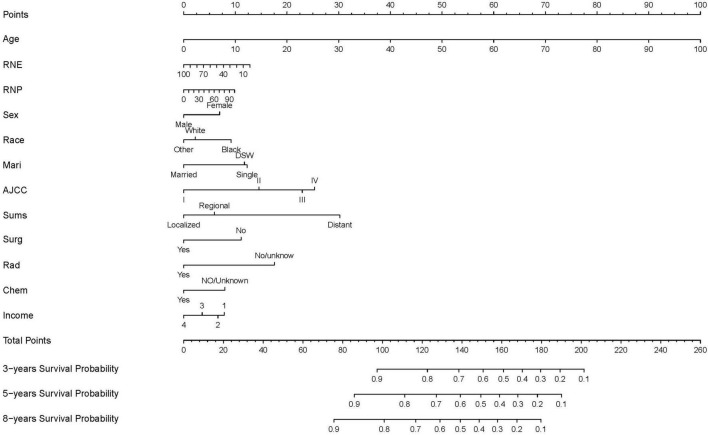
Nomogram predicting 3-, 5-, and 8-years CSS probability. Mari, marital status; Sums, summary of stage; Surg, surgery status; Rad, radiotherapy status; Chem, chemotherapy status; DSW, divorced, spereated, or widowed; income 1: <$35,000, $35,000–$44,999; income 2, $45,000–$59,999; income 3, $60,000–$74,999; income 4, $75,000+.

The finally constructed nomogram is shown in Figure 2. Based on the relevant factors stated above, the multiple regression model of the nomogram may be utilized to predict CSS probabilities. According to Figure 2, the summary stage had the greatest effect on survival rate, followed by AJCC stage, radiotherapy status, surgery status, chemotherapy status, race, sex, age at diagnosis, RNP, marital status, and income. Each component is represented as a line segment on the nomogram, and the numerical scale of the line defines the risk level presented by that factor. The sum of the scores for all of the criteria for each patient produces a total score that corresponds to their 3-, 5-, and 8-year CSS probabilities.

### Evaluating the nomogram using the validation cohort

**FIGURE 3:**
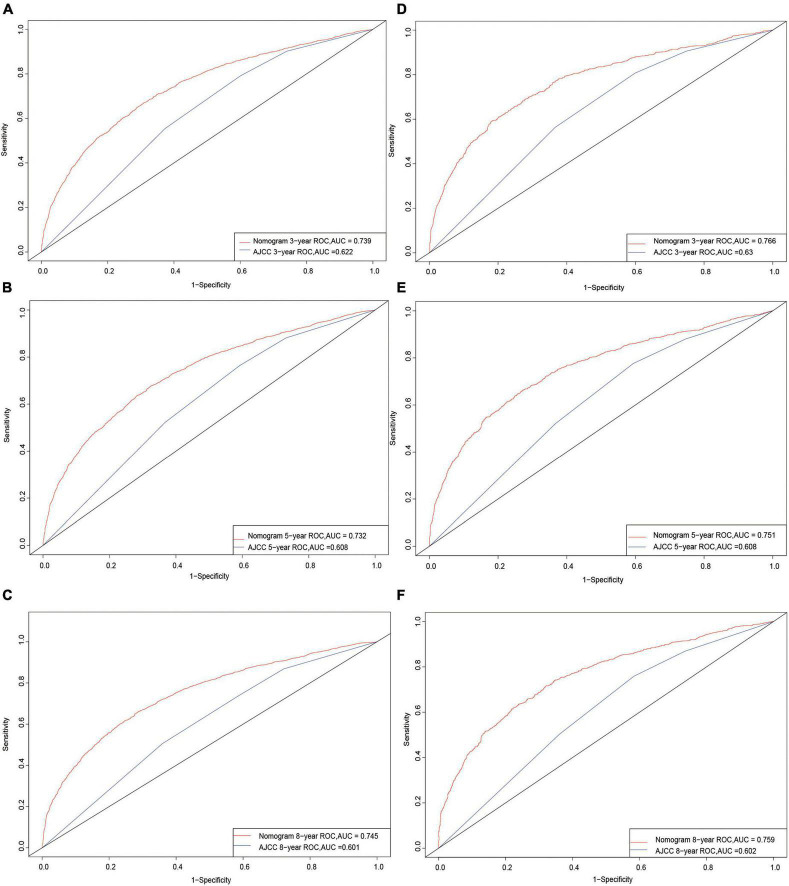
Receiver operating characteristic curves. The area under the ROC curve (AUC) for 3-, 5-, and 8-year CSS probability of the training cohort **(A–C)** and validation cohort **(D–F)**.

The C-index in the nomogram model was 0.710 for the training cohort and 0.725 for the validation cohort. The C-index for AJCC training cohort is 0.595, and validation cohort is 0.599. The new model’s C-index outranks the AJCC for training cohort by 0.115, and for validation cohort by 0.126. The AUCs at years 3, 5, and 8 were 0.739, 0.732, and 0.745, respectively, for the training cohort, and 0.766, 0.751, and 0.759 for the validation cohort (Figure 3).

The discrimination ability of the nomogram was evaluated using the NRI and IDI. The NRI values for the 3-, 5-, and 8-year CSS probabilities were 0.568 (95% confidence interval [CI] = 0.514–0.625), 0.581 (95% CI = 0.542–0.630), and 0.625 (95% CI = 0.587–0.682), respectively, for the training cohort, and 0.708 (95% CI = 0.623–0.796), 0.689 (95% CI = 0.612–0.762), and 0.748 (95% CI = 0.666–0.817) for the validation cohort. The IDI values for the 3-, 5-, and 8-year CSS probabilities were 0.125, 0.142, and 0.151, respectively, for the training cohort (*p* = 0.001), and 0.152, 0.158, and 0.168 for the validation cohort (*p* = 0.001).

**FIGURE 4:**
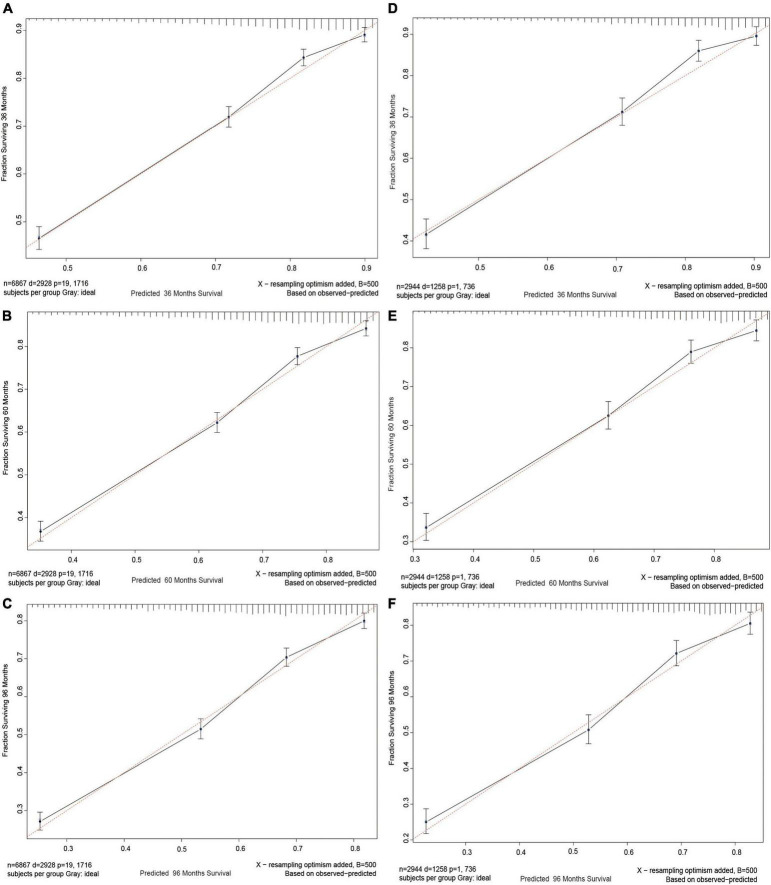
Calibration curves. Calibration curves for 3-, 5-, and 8-years CSS probability depict the calibration of each model in terms of the agreement between the predicted probabilities and observed outcomes of the training cohort **(A–C)** and validation cohort **(D–F)**.

In order to test between the real and ideal values of the model, the calibration plot was first utilized to confirm the discrimination ability of the model. The calibration plots of 3-, 5-, and 8-year CSS probabilities in the model were very close to the standard line (Figure 4), demonstrating that it had a high level of calibration.

**FIGURE 5:**
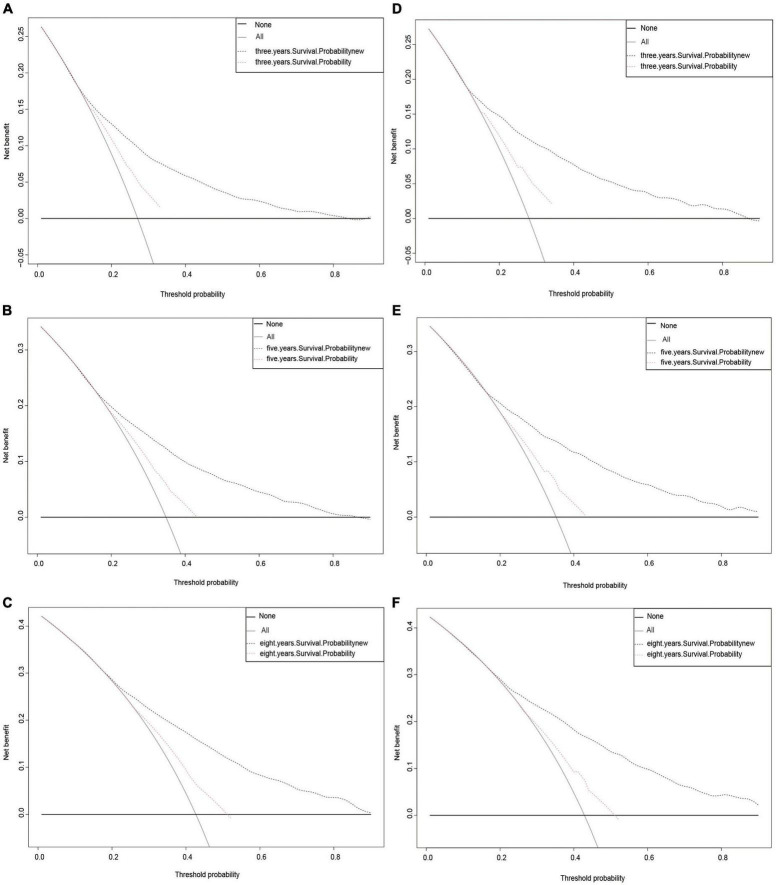
Decision curve analysis curves. Decision curve analysis of the training cohort **(A–C)** and validation cohort **(D–F)** for 3-, 5-, and 8-years CSS probability.

Finally, a DCA curve was drawn to demonstrate the clinical validity of the nomogram. The survival probability curves for the new model were all higher than those for the AJCC model (Figure 5), indicating that using the new method to predict 3-, 5-, and 8-year CSS probabilities is superior overall.

## Discussion

The newest guideline by the American Academy of Dermatology in 2018 paid more attention to individual factors in assessing NKLCSCC (19). Other classifications focus on molecular and immunohistochemical information, and biopsy techniques (20, 21). However, these current guidelines do not provide definite prognostic predictions for NKLCSCC. This study was therefore designed to analyze the combined prognostic factors for NKLCSCC in detail for the first time. Most of the published research studies have considered prognosis factors as an independent item (19, 21). Although most cSCCs can be successfully eradicated by surgical resection, cSCCs are somewhat characterized by high probabilities of recurrence, metastasis, and death. cSCC is most common in Caucasians and is more common in males than in females (with a 3:1 ratio) (22). Incidence increases with age, with an average age at diagnosis in the mid-60s (23), which differs from our results. All of these attributes indicate the need to develop a clinical prediction nomogram specific to NKLCSCC to assist doctors in making informed decisions. We were successful in creating a predictive nomogram that utilized the SEER database based on an integrated examination of demographic and clinicopathological factors. We then ascertained if the new model was superior to the AJCC staging system by comparing them.

The Cox regression results included in the nomogram indicate that besides summary stage, AJCC stage was the factor with the strongest effect on CSS probability. This was mostly due to the AJCC staging system including information on regional lymph node and distant metastases, which are very important prognostic factors in NKLCSCC (24). Summary stage as a factor only represents the metastasis of the patient, which had the highest HR. Among demographic characteristics, age has always been an important prognostic factor for tumors, and the present results were no exception. Black race also presented a worse prognosis than white or other race, and being married had a better prognosis than being single or divorced, separated, or widowed (DSW), which was consistent with the findings of other studies (25, 26).

While the incidence of NKLCSCC was previously found to be higher in males than in females (27), the sex distribution in the present study was relatively even. A particularly interesting aspect of this study was that previous studies have not investigated the impact of marital status on NKLCSCC prognosis; this study found that being single or DSW were risk factors for the prognosis. Regarding clinicopathological features, AJCC staging is known to influence treatment selection, outcome, and prognosis. We found that summary stage significantly affected the NKLCSCC CSS probability in a similar way to RNP, and a larger RNP increased the effect on the prognosis. As can be seen from Figure 2, surgery, radiotherapy, and chemotherapy treatments were also significant prognostic factors.

Prior to actually applying the model, we performed a number of assessments that are essential for clinical prediction models after constructing the nomogram and accounting for the indicated prognostic factors. First, we verified the discrimination ability of the model. The conventional ROC curve is a simple approach (28), and Figure 3 displays a nomogram with an AUC of >0.7. This illustrates the good overall discrimination capability of the nomogram. Comparing the ROC of the new model with that of the AJCC model also revealed that the new model outperformed it. Second, the C-index is a more generalized measure of the discrimination ability of prediction models between different outcomes for survival data (29). The current findings further demonstrate the outstanding discrimination ability of our new model. NRI is frequently used to compare the accuracy of the predictive capability of two models, and in contrast to AUC and the C-index, it quantifies the number of subjects correctly characterized by two models at a given set of cut-off points (30). In the training cohort, NRI values indicated increases in the proportion of correctly classifying CSS probabilities at 3, 5, and 8 years of 56.8, 58.1, and 62.4%, respectively, while those for the validation cohort were 70.8, 68.9, and 74.8% (*p* = 0.001). IDI is another metric that accounts for different cut-off points and can be used to reflect the overall improvement of a model, complementing NRI to a certain extent (31). The present IDI values demonstrated that, when compared with the AJCC model, the new model had better predictive power for 3-, 5-, and 8-year CSS probabilities by 12.5, 14.2, and 15.1% in the training cohort, respectively, and by 15.2, 15.8, and 16.8% in the validation cohort (*p* = 0.001).

The aforementioned four indicators unmistakably demonstrate the strong discrimination ability of the nomogram and that the model is capable of accurately predicting the survival rate of patients with NKLCSCC. We also drew calibration plots to assess the calibration accuracy of the model. In Figure 4, the calibration curve of the model demonstrates a uniform distribution and is fairly near to the standard line. This suggested that the model-predicted incidence was very close to the actual incidence, indicating that the model has good conformity. The model demonstrated a good performance level for estimating 3-, 5-, and 8-year CSS probabilities in patients with NKLCSCC when combined with the examination of discrimination and calibration abilities.

Finally, we assessed how effectively the model performed in healthcare situations. DCA is increasingly being used by researchers to evaluate the overall value of medical treatments for patients (17, 32). Our new approach had a better overall net benefit than the AJCC staging system and greater tolerance of survival probability, as seen in Figure 5. This indicates that the new model will provide patients with a greater overall benefit and assist physicians in making more informed treatment decisions.

Naturally, there were certain limitations to this study. First, we used the SEER database to collect analytic data in a retrospective manner, which could have induced some information bias. Second, several genetic markers, biomarkers, behavioral patterns, and other characteristics were not included in the study. Future cohort studies should more precisely pinpoint key prognostic markers and concentrate on including more prognostic factors and evaluating the model in external cohorts to provide more accurate findings.

## Conclusion

Based on a reasonably large retrospective population, we have developed the first nomogram for predicting 3-, 5-, and 8-year CSS probabilities in patients with NKLCSCC. This nomogram incorporates demographic and clinicopathological characteristics, and thorough validation and assessment make this model helpful and simple for doctors to utilize when making clinical decisions for specific patients. It was also demonstrated to provide beneficial recommendations. In the future, we hope to construct more-thorough nomograms based on a larger variety of data sources.

## Data availability statement

Publicly available datasets were analyzed in this study. This data can be found here: SEER database.

## Ethics statement

Approximately 34.6% of the U.S. population is represented in the population-based cancer registries used by SEER to gather data on cancer incidence. Each spring, based on information provided by the registry in November of the previous year, SEER releases a standardized set of research data. The database utilized for this study did not contain any personal identities and only comprised publically available data, therefore institutional review board permission was not necessary. These were acquired *via* the SEER*Stat software after receiving further clearances.

## Author contributions

All authors listed have made a substantial, direct, and intellectual contribution to the work, and approved it for publication.

## Footnotes

1
http://www.r-project.org
